# Medium-Chain Fatty Acids, Beta-Hydroxybutyric Acid and Genetic Modulation of the Carnitine Shuttle Are Protective in a *Drosophila* Model of ALS Based on TDP-43

**DOI:** 10.3389/fnmol.2018.00182

**Published:** 2018-05-31

**Authors:** Ernesto Manzo, Abigail G. O'Conner, Jordan M. Barrows, Dakotah D. Shreiner, Gabriel J. Birchak, Daniela C. Zarnescu

**Affiliations:** ^1^Department of Molecular and Cellular Biology, University of Arizona, Tucson, AZ, United States; ^2^Department of Neuroscience, University of Arizona, Tucson, AZ, United States; ^3^Department of Neurology, University of Arizona, Tucson, AZ, United States

**Keywords:** TDP-43, amyotrophic lateral sclerosis, metabolomics, lipid metabolism, beta lipid oxidation, carnitine shuttle

## Abstract

ALS patients exhibit dyslipidemia, hypermetabolism and weight loss; in addition, cellular energetics deficits have been detected prior to denervation. Although evidence that metabolism is altered in ALS is compelling, the mechanisms underlying metabolic dysregulation and the contribution of altered metabolic pathways to disease remain poorly understood. Here we use a *Drosophila* model of ALS based on TDP-43 that recapitulates hallmark features of the disease including locomotor dysfunction and reduced lifespan. We performed a global, unbiased metabolomic profiling of larvae expressing TDP-43 (wild-type, TDP^WT^ or disease-associated mutant, TDP^G298S^) and identified several lipid metabolism associated alterations. Among these, we found a significant increase in carnitine conjugated long-chain fatty acids and a significant decrease in carnitine, acetyl-carnitine and beta-hydroxybutyrate, a ketone precursor. Taken together these data suggest a deficit in the function of the carnitine shuttle and reduced lipid beta oxidation. To test this possibility we used a combined genetic and dietary approach in *Drosophila*. Our findings indicate that components of the carnitine shuttle are misexpressed in the context of TDP-43 proteinopathy and that genetic modulation of CPT1 or CPT2 expression, two core components of the carnitine shuttle, mitigates TDP-43 dependent locomotor dysfunction, in a variant dependent manner. In addition, feeding medium-chain fatty acids or beta-hydroxybutyrate improves locomotor function, consistent with the notion that bypassing the carnitine shuttle deficit is neuroprotective. Taken together, our findings highlight the potential contribution of the carnitine shuttle and lipid beta oxidation in ALS and suggest strategies for therapeutic intervention based on restoring lipid metabolism in motor neurons.

## Introduction

Amyotrophic lateral sclerosis (ALS) is a complex neurodegenerative disease characterized by upper and lower motor neuron atrophy. Motor neuron dysfunction leads to rapid muscle loss, weakness, paralysis, and eventual death (Al-Chalabi et al., 2012; Robberecht and Philips, 2013). There is no cure for ALS and the available treatments are at best palliative. One of the pathological hallmarks of disease is the accumulation of the TAR DNA binding protein 43 (TDP-43) in cytoplasmic aggregates, both in glial and neuronal cells (Neumann et al., 2006). These aggregates have been identified in 97% of all ALS, and 45% of frontotemporal degeneration (FTD) cases making it an important, unifying feature of the ALS/FTD spectrum disorder (Ling et al., 2013).

Clinical observations indicate that in addition to loss of motor function, ALS patients exhibit gross metabolic changes that include weight loss, hypermetabolism (in > 50% patients) (Desport et al., 2005; Bouteloup et al., 2009), insulin resistance (Wuolikainen et al., 2016), and impaired glucose tolerance (Pradat et al., 2010). What remains unclear is how these metabolic changes arise, or may contribute to disease progression. Indeed, key metabolic alterations, such as weight loss, are undoubtedly influenced by loss of muscle mass due to degeneration of motor neurons. However, work in ALS animal models has demonstrated that defects in cellular energetics precede denervation and muscle atrophy (reviewed in Dupuis et al., 2011). In addition, recent clinical studies in patients have shown that high caloric diets can improve disease outcomes suggesting that metabolic intervention may provide effective therapeutic strategies (Wills et al., 2014). Moreover, metabolic profiling data from ALS patients show significantly altered metabolites consistent with defects in cellular energetics (Lawton et al., 2012, 2014). These findings highlight the need for identifying metabolic alterations at the cellular level, in motor neurons and glia, that may uncover key strategies for preventing and ameliorating metabolic dysregulation in ALS.

We have previously developed a *Drosophila* model of ALS based on neuronal or glial expression of human TDP-43 (Estes et al., 2011, 2013). TDP-43 overexpression in either motor neurons or glial cells leads to reduced locomotor function, reduced lifespan, and synaptic alterations at the neuromuscular junction (Coyne et al., 2014, 2017). Here, we use this *Drosophila* model of ALS to dissect motor neuron and glia specific alterations in metabolism and probe the role of the carnitine shuttle and lipid beta oxidation in disease. Metabolic profiling of larvae expressing TDP-43 (wild-type or disease associated mutant G298S) in motor neurons identifies an accumulation of carnitine conjugated long-chain fatty acids and a significant reduction in carnitine and beta-hydroxybutyrate, a ketone precursor. Together with transcriptional profiling data, these metabolic changes point to defects in the carnitine shuttle, which is required for long-chain fatty acid import into mitochondria and subsequent breakdown by lipid beta oxidation ultimately leading to ATP production. We reasoned that since medium-chain fatty acids and beta-hydroxybutyrate can cross into the mitochondrial matrix independent of the carnitine shuttle, TDP-43 dependent phenotypes might be attenuated by dietary supplementation with these molecules. In this study, we demonstrate that these dietary interventions mitigate locomotor defects caused by TDP-43 in motor neurons. Furthermore, genetic manipulations of carnitine shuttle components modulate locomotor function in TDP-43 expressing larvae. Taken together, these findings provide support to the notion that improving cellular energetics by countering metabolic alterations such as carnitine shuttle deficits in motor neurons can mitigate ALS phenotypes.

## Materials and methods

### *Drosophila* genetics

All *Drosophila* stocks and crosses were kept on standard yeast/cornmeal/molasses food at 25°C except for genetic interaction experiments where D42 > TDP-43 crosses were maintained at 22°C to facilitate increased survival of adult males needed for crossing with CPT mutant females. Transgenics harboring human TDP-43 human wild type (TDP^WT^) and disease-associated mutant (TDP^G298S^) variants were previously described (Estes et al., 2011, 2013). TDP-43 was expressed in motor neurons and glia using the D42 GAL4 (Gustafson and Boulianne, 1996) and repo GAL4 (Sepp et al., 2001) drivers, respectively. For controls, w^1118^ was crossed with either the D42 or repo Gal4 drivers, as appropriate. To manipulate CPT1 (*withered, whd*) and CPT2, we used: *y*^1^*v*^1^*; P{y[*+*t7.7] v[*+*t1.8]* = *TRiP.HMS00040} attP2/TM3, Sb* and *w*^1118^; *PBac{w[*+*mC]* = *WH} CPT2*^*f*02667^*/TM6B, Tb*^1^ (from the Bloomington Stock Center). *y*^1^ *v*^1^*; P{y[*+*t7.7]* = *CaryP} attP2* was used as a genetic background control for CPT1 RNAi knock-down experiments.

To evaluate RNAi knock-down efficiency of CPT1 RNAi, *y*^1^*v*^1^*; P{y[*+*t7.7] v[*+*t1.8]* = *TRiP.HMS00040} attP2/TM3, Sb1was* crossed with the ubiquitous driver actin GAL4 (w; Actin GAL4/CyO, from the Bloomington Stock Center). Whole flies expressing CPT1 RNAi ubiquitously were isolated, and CPT1 mRNA levels were tested by qPCR (see below for qPCR protocol and primers). *CPT2*^*f*02667^ mutants are homozygous lethal, therefore, we introduced this allele as a heteryzgote in the context of TDP-43. CPT2 reduction was evaluated by qPCR in adult heads *elav*^*c*155^ *GAL4*/+; +/+; *CPT2*^*f*02667^/+.

### Fly food supplementation

Standard yeast/cornmeal/molasses food was heated and then allowed to cool to 55–60°C. The food was supplemented with either coconut oil [organic, expeller pressed unrefined virgin coconut oil (365 everyday value, Whole Foods market)], ethyl heptanoate (Sigma cat#: W243728) or sodium R-,S-beta-hydroxybutyrate (BHB, Sigma cat#: H6501). Following supplement addition to the desired concentration, the food was dispensed into vials and allowed to cool.

### Locomotor assays

Larval turning assays were performed as previously described (Estes et al., 2011, 2013). Briefly, third instar larvae were placed at room temperature on grape juice plates made of 2.5% agar and grape juice. Larvae are allowed to acclimate on the grape plate, then gently turned ventral side up. Larvae were individually timed from the time they are turned to the time they reorientate themselves ventral side down and make their first forward motion. The number of larvae tested in each experiment has been noted in the appropriate Results section. Larval turning data were analyzed using Kruskal-Wallis multiple comparison tests (GraphPad Prism v7).

### Metabolite analysis

Metabolomic analysis was conducted by Metabolon as previously described (Joardar et al., 2014). Briefly, D42-Gal4 virgin females were crossed with UAS-TDP-43-YFP or w^1118^ males and raised on standard food. 50–60 wandering third instar larvae (50–60 mg) per sample were collected and flash-frozen in liquid nitrogen. Protein from larval samples (*N* = 5 replicates per genotype) was precipitated with methanol under vigorous shaking conditions, and processed using ultrahigh performance liquid chromatography/mass spectrometry (UHPLC/MS), or by gas chromatography/mass spectrometry (GC/MS). Following normalization to Bradford protein concentration, log transformation and imputation of missing values, if any, with the minimum observed value for each compound, One-way ANOVA was used to identify biochemicals that differed significantly between experimental groups.

### RNA isolation, cDNA preparation, and qPCR

Ventral nerve cords (VNCs) were dissected from five wandering third instar larvae per genotype, per biological replicate. Dissections were performed in ice cold HL-3 saline buffer (70 mM NaCL, 5 mM KCl, 22 mM MgCl_2_, 10 mM NaHCO_3_, 5 mM trehalose, 115 mM sucrose, 5 mM HEPES, pH 7.3). Briefly, dissections were performed to isolate both brain lobes and ventral nerve cord where motor neurons and glia reside. VNCs were then transferred to 50 μL RLT Buffer (Qiagen) with 1% BME before being homogenized and stored at −80°C. RNA was extracted using the RNeasy RNA Extraction Kit (Qiagen) and stored at −80°C. Genomic DNA (gDNA) was cleared using a DNase I (Thermo Fisher Scientific) digestion protocol. Following gDNA clearing, reverse transcription was performed to generate complement DNA (cDNA) from mRNA using the Maxima First Strand cDNA Synthesis Kit for RT-qPCR (Thermo Fisher Scientific). The quality of the cDNA was tested using GPDH primers (see below for sequence) and GoTaq endpoint polymerase chain reaction (PCR) protocol (Promega). Quantitative PCR was performed using a SYBR Green protocol (Applied Biosystems) and primers designed to span exon-exon junctions, with the exception of *colt*, which has only one exon and was assayed using the TaqMan gene expression assay (Thermo Fisher). Each reaction was conducted in 3-6 biological replicates with three technical replicates each.

### Primer sequences and taqman probes

*CPT1/withered (whd)*: Forward primer—AGGAACTGCAGCCTATCATGG; Reverse primer - GGAGTTGCTTTGCCCTTCAG

*CPT2*: Forward primer—GTGCCACTTGGTGTTCTGAC; Reverse primer—TGTCGAACCAACGATTCTGC

*GPDH:* Forward primer—CCGCAGTGCTTGTTTTGCT*;* Reverse primer—TATGGCCGAACCCCAGTTG

*CACT*/*congested-like trachea (colt)* : Taqman - Dm0180 8195_s1

*GPDH:* Taqman - Dm01841185_m1

### Statistics

#### Mass spectrometry

**Figure 1:**
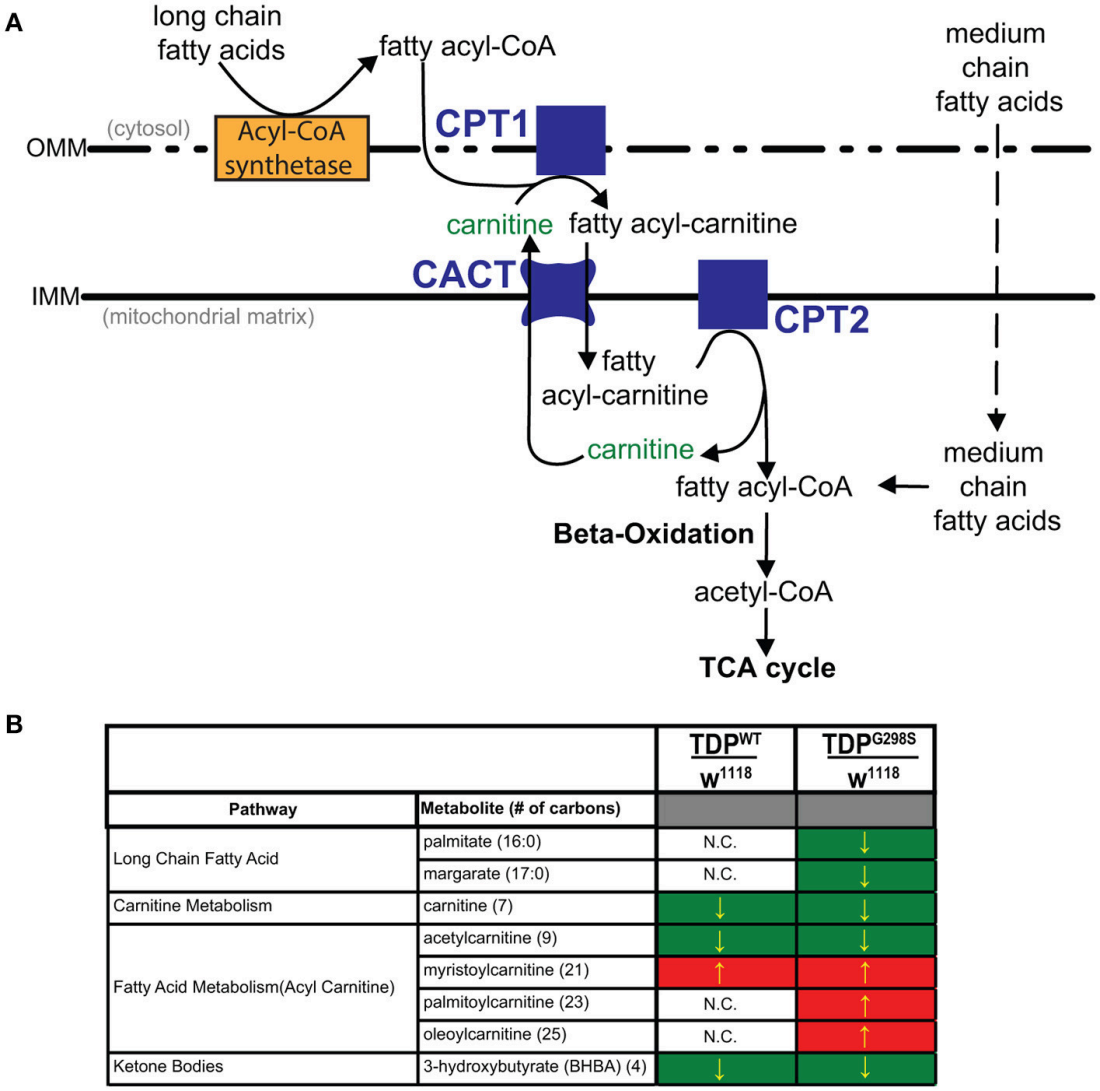
Metabolomic profiling of ALS larvae uncovers deficits in carnitine, carnitine conjugation and lipid beta oxidation. **(A)** Carnitine shuttle components and fatty acid import into mitochondria. CPT1, 2, carnitine acyltransferases; CACT, carnitine acylcarnitine transferase; IMM, Inner mitochondrial membrane; OMM, Outer mitochondrial membrane. **(B)** Summary of significantly altered metabolites in TDP^WT^ and TDP^G298S^ compared to w^1118^ controls. Green indicates significant downregulation and red indicates significant upregulation (*P*_value_ < 0.05).

One-way ANOVA was used to identify metabolites that differed significantly between experimental groups. A summary of the numbers of metabolites that achieved statistical significance (*P*_value_ ≤ 0.05) is reported (Figure 1, Table 1). The false discovery rate (Q value) was calculated to take into account the multiple comparisons that normally occur in metabolomic-based studies (see Table 1 and Storey and Tibshirani, 2003).

**Table 1:** Summary of metabolites associated with lipid metabolism in TDP^WT^ and TDP^G298S^ compared to w^1118^ controls.

		$\frac{TDP^{WT}}{w^{1118}}$			$\frac{TDP^{G298S}}{w^{1118}}$		
**Sub pathway**	**Biochemical name (number of carbons)**		***P-*value**	***Q-*value**		***P-*value**	***Q-*value**
Medium chain fatty acid	Caprylate (8:0)	0.91	0.322	0.5054	0.84	0.0947	0.0743
	Caprate (10:0)	1.13	0.1394	0.3364	1.03	0.6364	0.2714
	10-undecenoate (11:1n1)	1	0.881	0.6922	0.94	0.6643	0.2776
	Laurate (12:0)	1.12	0.2719	0.475	1.06	0.4854	0.2304
	5-dodecenoate (12:1n7)	0.88	0.2037	0.4147	0.82	0.0508	0.048
Long chain fatty acid	Myristate (14:0)	1.03	0.7417	0.6697	0.97	0.9668	0.3551
	Myristoleate (14:1n5)	1.02	0.7413	0.6697	1.01	0.7832	0.3091
	Pentadecanoate (15:0)	0.92	0.7983	0.6746	0.77	0.1489	0.1005
	Palmitate (16:0)	0.91	0.4195	0.5696	0.79	0.0435	0.0433
	Palmitoleate (16:1n7)	0.97	0.998	0.7217	0.82	0.2499	0.1445
	Margarate (17:0)	0.76	0.2025	0.4147	0.65	0.0378	0.0396
	10-heptadecenoate (17:1n7)	0.85	0.7824	0.6746	0.68	0.23	0.1364
	Oleate (18:1n9)	0.92	0.7135	0.662	0.77	0.1768	0.112
	Nonadecanoate (19:0)	0.89	0.6763	0.6599	0.9	0.7703	0.307
	10-nonadecenoate (19:1n9)	0.7	0.2182	0.4229	0.73	0.388	0.1938
	7-nonadecenoate (19:1n12)	0.82	0.4783	0.5832	0.62	0.0979	0.0762
	Arachidate (20:0)	0.85	0.4454	0.5755	0.74	0.1622	0.1063
	Eicosenoate (20:1n9 or 11)	0.85	0.6873	0.662	0.7	0.2915	0.1609
	Erucate (22:1n9)	0.77	0.3719	0.5294	0.71	0.219	0.1332
Carnitine metabolism	Carnitine (7)	0.88	0.0035	0.0411	0.86	0.0006	0.0019
Fatty acid metabolism (acyl carnitine)	Acetylcarnitine (9)	0.83	0.01	0.0813	0.74	0.0001	0.0007
	Myristoylcarnitine (21)	1.41	0.0314	0.1641	1.99	0.0001	0.0007
	Palmitoylcarnitine (23)	1.14	0.3647	0.5246	1.41	0.0352	0.038
	Stearoylcarnitine (25)	0.82	0.2075	0.4148	0.93	0.6305	0.2695
	Linoleoylcarnitine (25)	0.95	0.9568	0.7192	1.42	0.0723	0.0612
	Oleoylcarnitine (25)	1.29	0.1606	0.3604	1.55	0.0176	0.0221
Ketone bodies	3-hydroxybutyrate (BHBA) (4)	0.63	0.0137	0.0988	0.56	0.0012	0.003

*Altered metabolites in third instar larvae crossed with the motor neuron driver D42 GAL4 were measured using gas or liquid chromatography followed by mass spectrometry. Each biochemical raw output was rescaled to set the median equal to 1. Values for each sample are normalized by Bradford protein concentration. Red and green colored cells indicate statistically significant changes (P-value < 0.05) that are increased and decreased, respectively. Light red and light green colored cells indicate upward or downward trends, respectively (P-value < 0.1). Q-values represent the false discovery rate*.

#### RT-qPCR

CT values were averaged and analyzed using the ΔΔCT method (Pfaffl, 2001) to normalize target gene values to those of GPDH (housekeeping gene), and to compare relative transcript levels between TDP-43^WT^ or TDP-43^G298S^ and w^1118^ controls. The Kruskal-Wallis test was used to assess statistical significance in GraphPad Prism v7.

#### Larval turning

To calculate significance we used the non-parametric Kruskal-Wallis test which assumes non-equal variance between data sets.

## Results

### Lipid metabolism is dysregulated in a *Drosophila* model of ALS based on TDP-43

To gain insights into ALS related metabolic defects, we took an unbiased, global metabolomics approach (see Materials and methods, and Joardar et al., 2014). In brief, whole larvae expressing TDP-43 (either TDP^WT^ or TDP^G298S^) in motor neurons with the D42 GAL4 driver were analyzed by Metabolon, Inc. using ultrahigh performance liquid chromatography-tandem mass spectroscopy (UPLC-MS/MS) and gas chromatography-mass spectroscopy (GC-MS). Among the 133 compounds associated with lipid metabolism that were detected by the Metabolon platform, we found 21 metabolites significantly altered in the context of TDP^WT^ (*P* < 0.05) and 31 altered in the context of TDP^G298S^ (*P* < 0.05) (see Supplemental Table 1). More specifically, we found an increase in fatty acid carnitine conjugates (e.g., myristoylcarnitine, palmitoylcarnitine, oleoylcarnitine, and linoleoylcarnitine). These were more prominent in TDP^G298S^ but were also detected in TDP^WT^ larvae, suggesting decreased lipid beta-oxidation (see Figure 1 and Table 1). Consistent with these observations, the levels of carnitine, which is required for mitochondrial entry of long-chain fatty acids, are significantly decreased. In addition, beta-hydroxybutyrate, a ketone precursor and a key product of lipid beta-oxidation, is decreased in both TDP^WT^ and TDP^G298S^ expressing larvae. These findings are consistent with alterations in the mitochondrial carnitine shuttle and decreased mitochondrial lipid metabolism in the context of TDP-43 proteinopathy.

### Transcriptional profiling indicates TDP-43 dependent alterations in the expression of carnitine shuttle components CPT1, CPT2, and CACT

**Figure 2:**
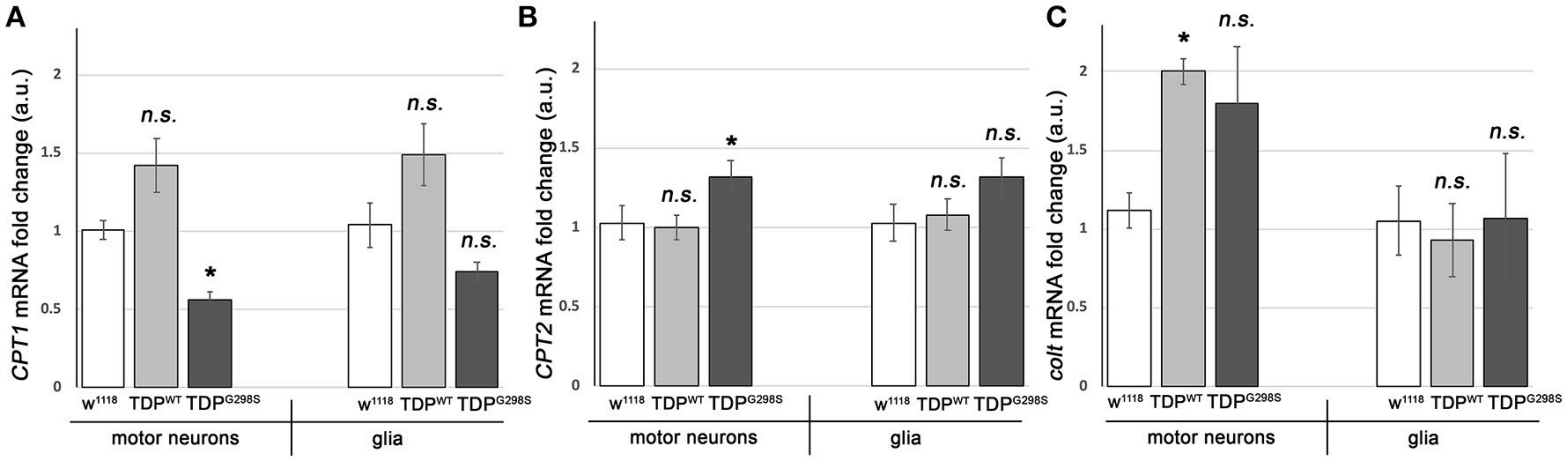
TDP-43 causes alterations in the expression of carnitine shuttle components. **(A–C)** Transcriptional profiling using q RT PCR of *CPT1* mRNA **(A)**, *CPT2* mRNA **(B)** and *colt* mRNA **(C)** indicates TDP-43 dependent changes in motor neurons and glia. TDP^WT^ or TDP^G298S^ were expressed using D42 GAL4 (motor neurons) or repo GAL4 (glia). Kruskal-Wallis multiple comparisons test was used to calculate significance. n.s., not significant; ^*^P_value_ < 0.05.

To further probe the integrity of the carnitine shuttle in the context of TDP-43 proteinopathy, we used quantitative PCR (qPCR) to measure the expression of its key components, CPT1, CPT2, and CACT (Figure 2). TDP^WT^ or TDP^G298S^ were expressed in motor neurons and glia with D42 GAL4 and repo GAL4, respectively (Estes et al., 2013). VNCs comprising the majority of motor neurons, interneurons and glia expressing TDP-43 as well as w^1118^ controls were dissected from wandering third instar larvae. Following RNA isolation and cDNA preparation, qPCR was performed using primers specific to *Drosophila CPT1, CPT2*, and *congested-like trachea* (*colt*, CACT homolog) transcripts (see Materials and Methods for experimental details and primer information). These experiments revealed complex changes in the expression of carnitine shuttle components in motor neurons and glia, in a variant dependent manner (see Figure 2). When TDP^WT^ was expressed in motor neurons we found *colt* to be significantly upregulated [2.00 (*N* = 3) fold change, *P*_value_ = 0.04], while *CPT1* trended upwards [1.42 (*N* = 6) *P*_value_ = 0.08] and *CPT2* remained unaltered (*N* = 6). When expressed in glial cells, there were no changes detected in *CPT1, CPT2* or *colt* expression. TDP^G298S^ expression in motor neurons caused a significant decrease in *CPT1* [0.56-fold (*N* = 6), *P*_value_ = 0.04], an increase in *CPT2* [1.3-fold (*N* = 6), *P*_value_ = 0.04] and an upward trend in *colt* [1.79-fold (*N* = 3), *P*_value_ = 0.14]. Although additional studies are needed to precisely determine the mechanism by which long-chain fatty acid import shuttling is altered by TDP^WT^ and disease associated TDP^G298S^, our data support the notion that components of the carnitine shuttle are affected at the transcriptional level in the context of TDP-43 proteinopathy.

### Key components of the carnitine shuttle modulate TDP-43 dependent phenotypes

**Figure 3:**
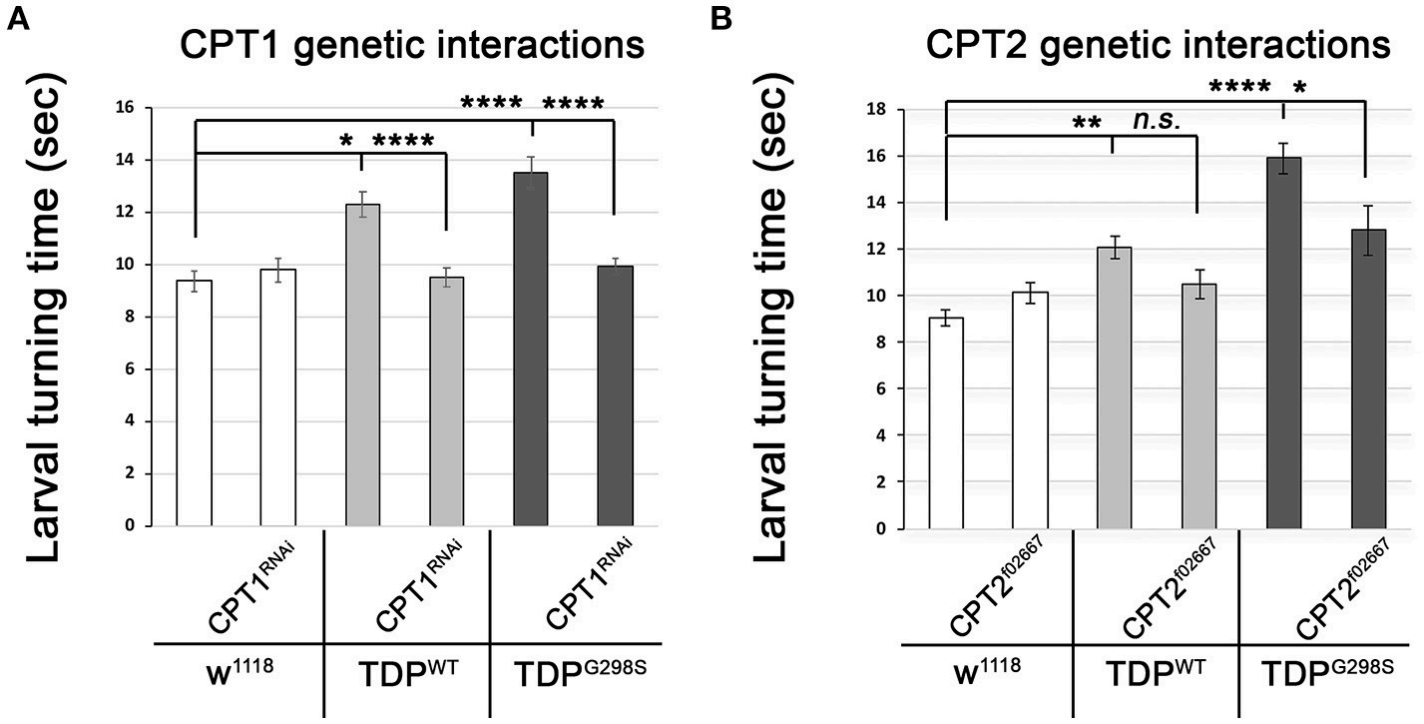
Genetic interaction experiments show that CPT1 and CPT2 mitigate locomotor dysfunction caused by TDP^WT^ and TDP^G298S^, in a variant dependent manner. **(A)** CPT1 knock-down by RNAi (CPT1^RNAi^, using y^1^v^1^; P{y[+t7.7] v[+t1.8] = TRiP.HMS00040}attP2/TM3, Sb^1^) mitigates TDP^WT^ and TDP^G298S^ larval turning times. **(B)** CPT2 loss of function (CPT2^f02667^, using w^1118^; PBac{w[+mC]=WH}CPT2^f02667^/TM6B, Tb^1^) mitigates TDP^G298S^ larval turning times. TDP^WT^ or TDP^G298S^ were expressed in motor neurons using D42 GAL4. Genotypes as indicated. Kruskal-Wallis multiple comparisons test was used to calculate significance. ^*^P_value_ < 0.05, ^**^P_value_ < 0.01, ^****^P_value_ < 0.0001.

The TDP-43 dependent transcriptional changes we found in *CPT1* and *CPT2* predict that dosage alterations in these genes modify ALS-like phenotypes in our model. Indeed, we found that knocking down *CPT1* (*withered, whd* in *Drosophila*) by RNAi causes a reduction in larval turning time from 12.3 ± 0.48 s (*N* = 30) to 9.51 ± 0.37 s (*N* = 30) (*P*_value_ < 0.0001) in the context of TDP^WT^ and from 13.53 ± 0.59 s to 9.95 ± 0.32 s (*N* = 30) in the context of TDP^G298S^ (*P*_value_ < 0.0001) but has no effect on its own (*N* = 30) (Figure 3A). *CPT1* levels were reduced by 70% in whole flies expressing *CPT1* RNAi using the ubiquitous actin GAL4 driver (see Materials and Methods and data not shown). These genetic interaction results are consistent with the *CPT1* transcript trending upwards in the context of TDP^WT^ overexpression in motor neurons (compare Figure 3A with Figure 2A). For mutant TDP-43, the rescue by *CPT1* RNAi together with our qPCR findings that *CPT1* transcript is downregulated in motor neurons expressing TDP^G298S^ suggests that *CPT1* downregulation in the context of TDP^G298S^ is a compensatory mechanism (compare Figure 3A with Figure 2A). Similarly, reducing *CPT2* dosage using a loss of function allele (*CPT2*^*f*02667^, see Materials and Methods) mitigates TDP^G298S^ dependent locomotor dysfunction (*N* = 21), as expected from our findings that *CPT2* mRNA is upregulated by TDP^G298S^ overexpression in motor neurons (compare Figure 3B with Figure 2B). Consistent with our transcriptional profiling data, reducing *CPT2* dosage has no effect on larval turning in w^1118^ controls (*N* = 24) or larvae expressing TDP^WT^ in motor neurons (*N* = 27). qPCR analyses of *CPT2* knock-down using the *CPT2*^*f*02667^ allele shows a 35% reduction in *CPT2* mRNA (see Materials and Methods and data not shown). Taken together these results indicate that at least some aspects of TDP-43 toxicity are caused by alterations in specific carnitine shuttle components, and manipulating their expression genetically mitigates locomotor dysfunction in our *Drosophila* model of ALS.

### Dietary supplementation using medium-chain fatty acids or beta-hydroxybutyrate mitigates locomotor defects caused by TDP-43 overexpression in motor neurons

**Figure 4:**
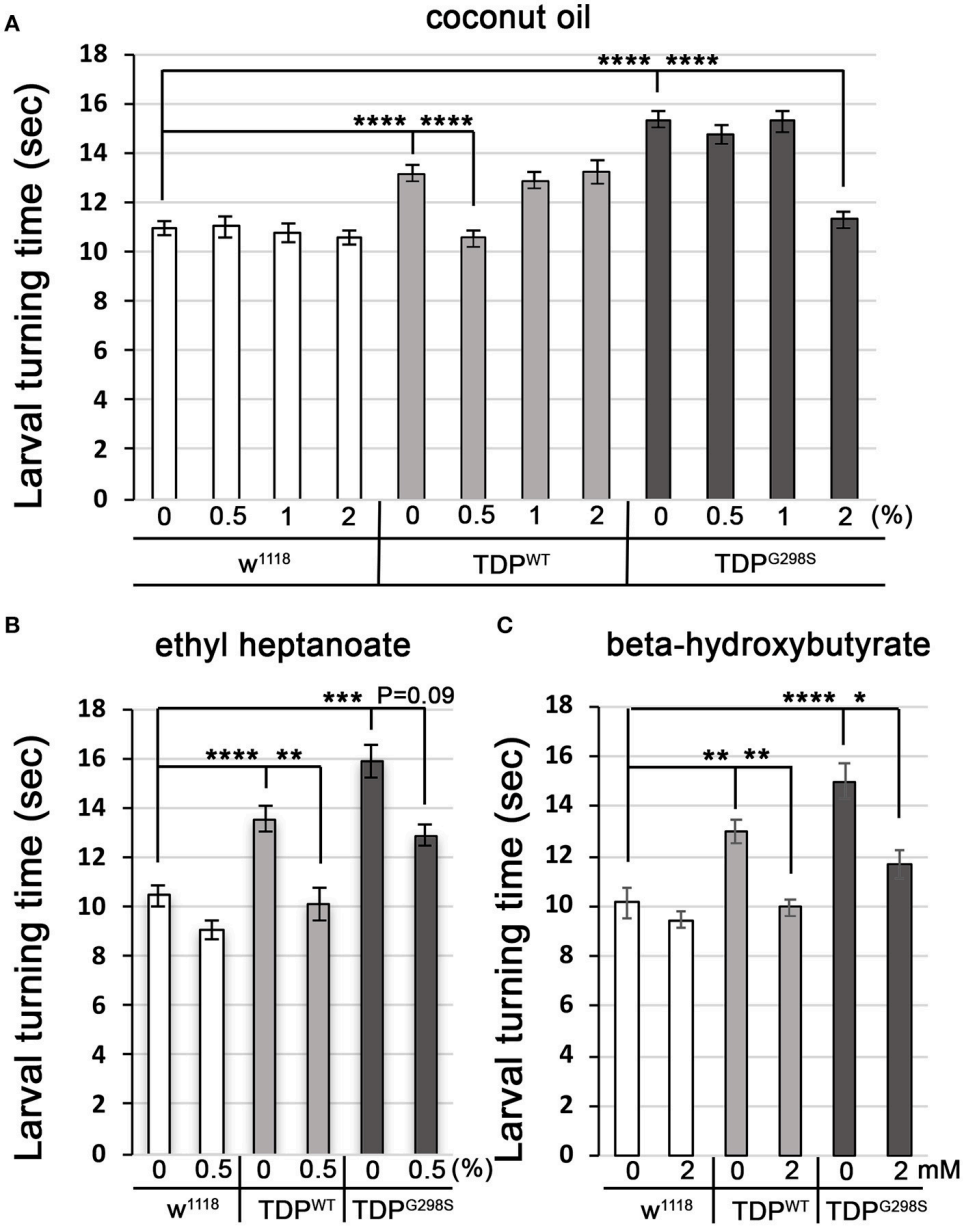
Medium-chain fatty acids and ketones mitigate TDP-43 dependent locomotor dysfunction in a *Drosophila* model of ALS. **(A,B)** Larval turning times, used to measure locomotor deficits caused by TDP^WT^ or TDP^G298S^, are rescued by feeding different medium-chain fatty acids [coconut oil **(A)** or ethyl heptanoate **(B)**]. **(C)** Ketone (beta-hydroxybutyrate) feeding reduces larval turning times in ALS larvae. TDP^WT^ or TDP^G298S^ were expressed in motor neurons using D42 GAL4. Genotypes and treatments as indicated. Kruskal-Wallis multiple comparisons test was used to calculate significance. ^*^P_value_ < 0.05, ^**^P_value_ < 0.01, ^***^P_value_ < 0.001, ^****^P_value_ < 0.0001.

Fatty acyl-CoA molecules are transported from the cytosol into the mitochondria for lipid beta oxidation through the carnitine shuttle system, which contains two carnitine acyltransferases (CPT1, on the outer mitochondrial membrane, and CPT2, on the inner mitochondrial membrane) and the carnitine acylcarnitine transferase (CACT) that shuttles a fatty acylcarnitine inside the mitochondrial matrix while a carnitine is shuttled back out (Figure 1A). These components are conserved in *Drosophila* (see Materials and Methods for fly specific gene names). Generally, long-chain fatty acids (13–21 carbons) depend on the carnitine shuttle while medium and short-chain-chain chain fatty acids (6–12 carbons and fewer) can cross the mitochondrial membranes freely (Rinaldo et al., 2002). Given the alterations in the carnitine shuttle identified by metabolic profiling, we reasoned that medium-chain fatty acids may be able to enter the mitochondria and provide neuroprotection. To test this possibility, we fed TDP-43 expressing larvae fly food supplemented with coconut oil, which contains a mixture of medium-chain fatty acids at varying concentrations, ranging from 0.5 to 2% (Figure 4A). These experiments showed that TDP-43 dependent locomotor defects are mitigated by 0.5% coconut oil for TDP^WT^ (13.2 ± 0.3 s on regular food (*N* = 143), vs. 10.5 ± 0.3 s on 0.5% coconut oil (*N* = 96), *P*_value_ < 0.0001) and 2% for TDP^G298S^ (15.3 ± 0.4 s on regular food (*N* = 145) vs. 11.3 ± 0.4 s on 2% coconut oil (*N* = 106), *P*_value_ < 0.0001). Notably, none of the tested coconut oil concentrations affected the locomotor function of w^1118^ control larvae (*N* = 87–122). While we do not know the basis for the observed variant dependent effect of coconut oil, these findings are consistent with the more severe lipid metabolism defects found in the context of TDP^G298S^.

Another medium-chain fatty acid that we tested is ethyl heptanoate, a modified version of heptanoate, a seven carbon fat previously used to successfully improve oxidative phosphorylation in a SOD1 mouse model (Tefera et al., 2016). The rationale for using this odd chain fatty acid is that it can bypass the carnitine shuttle, and upon beta oxidation mediated breakdown it generates acetyl-CoA that feeds directly into the TCA cycle and propionyl-CoA that can be utilized as an alternative fuel, also feeding into the TCA cycle (Borges and Sonnewald, 2012; Tefera et al., 2017). These experiments show that feeding 0.5% ethyl heptanoate significantly improves the locomotor function of TDP^WT^ larvae (from 13.5 ± 0.7 s on regular food (*N* = 81) to 10.1 ± 0.4 s (N=85), *P*_value_ < 0.01) (Figure 4B). Neither 1% nor 2% ethyl heptanoate had an effect on TDP^WT^ locomotion (*N* = 36–83, data not shown). For TDP^G298S^ larvae, neither 0.5, 1, or 2% ethyl heptanoate had a detectable effect on locomotor function (*N* = 42–99), possibly due to the narrow concentration range we tested. We note that when performing the Kruskall-Wallis test on TDP^G298S^ and w^1118^ controls only (ommiting TDP^WT^) the *P*_value_ for larval turning on 0.5% ethyl heptanoate reaches statistical significance (*P*_value_ = 0.03). No effect was detected in w^1118^ controls fed ethyl heptanoate (*N* = 49–83).

Interestingly, feeding 2 mM beta-hydroxybutyrate (BHB) improves locomotor function in both TDP^WT^ (12.99 ± 0.5 s on regular food (*N* = 30) vs. 9.97 ± 0.35 s on 2 mM BHB (*N* = 30), *P*_value_ < 0.01) and TDP^G298S^ larvae (15.02 ± 0.72 s on regular food (*N* = 30) vs. 11.68 ± 0.53 s on 2 mM BHB (*N* = 30), *P*_value_ < 0.05) while exerting no detectable effect on w^1118^ controls (*N* = 30) (Figure 4C). This concentration was chosen based on previous reports that BHB mitigates phenotypes in a *Drosophila* model of seizures (Li et al., 2017). Taken together, our findings support the notion that TDP-43 toxicity manifests at least in part through lipid oxidation defects, and bypassing the carnitine shuttle by providing medium-chain fatty acids or the ketone precursor BHB mitigates locomotor dysfunction in our *Drosophila* model of ALS. Although we only tested a limited range of concentrations our results indicate the neuroprotective potential of medium-chain fatty acids and the ketone precursor BHB. These data suggest a potentially promising dietary intervention that provides alternative fuels for improving oxidative phosphorylation and increasing ATP production as previously shown for SOD1 mice (Tefera et al., 2016).

## Discussion

**Figure 5:**
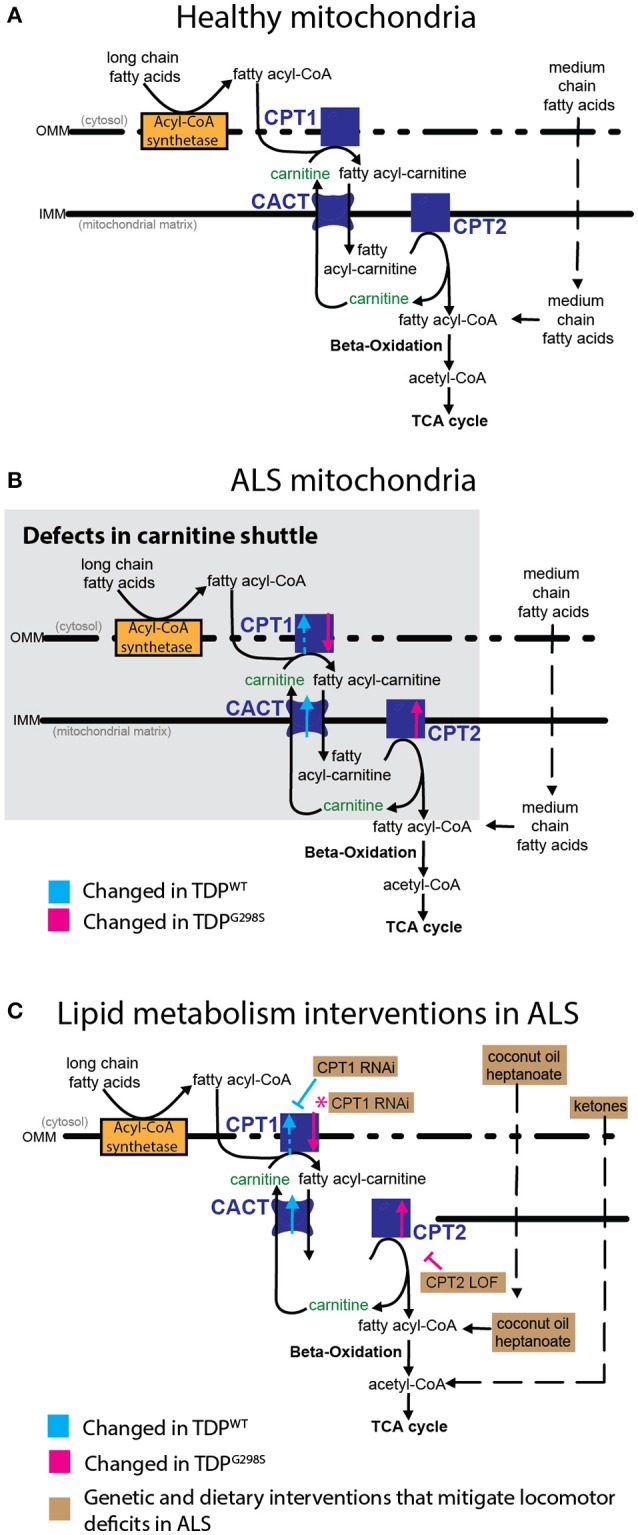
Model of mitochondrial dysfunction in ALS and proposed interventions. Expression of TDP^WT^ or TDP^G298S^ in motor neurons causes alterations in the expression of carnitine shuttle components (ALS mitochondria, **B**) compared to controls (Healthy mitochondria, **A**). Blue dashed line for CPT1 indicates an upward trend in transcript levels (*P*_value_ = 0.08). These defects can be countered genetically, by reducing CPT1 in the context of TDP^WT^ (inhibitory blue symbol), or reducing CPT2 in the context of TDP^G298S^ (inhibitory magenta symbol). Magenta asterisk indicates a potential compensatory mechanism whereby CPT1 RNAi knock-down mitigates TDP^G298S^ locomotor phenotypes despite CPT1 mRNA being reduced. Alternatively, dietary supplementation with medium-chain fatty acids or beta-hydroxybutyrate bypasses carnitine shuttle dysfunction leading to significant improvement in locomotor function (**C**, Lipid metabolism interventions in ALS).

Clinical observations of dyslipidemia, hypermetabolism and weight loss in ALS (reviewed in 26) together with findings that cellular energetics deficiency precedes denervation (reviewed in Dupuis et al., 2011) suggest that metabolic dysregulation correlates with and may contribute to disease progression. Here we investigated metabolic changes in a *Drosophila* model of ALS that recapitulates key features of the human disease including the presence of insoluble TDP-43 complexes, synaptic deficits, locomotor dysfunction and reduced lifespan (Estes et al., 2011, 2013; Coyne et al., 2014, 2017). Although our *Drosophila* model is based on TDP-43 overexpression, it remarkably predicts molecular alterations in ALS spinal cords (Coyne et al., 2014) or patient derived motor neurons (Coyne et al., 2017), as well as confirms the limited neuroprotective potential of pioglitazone as a therapeutic for ALS (Joardar et al., 2014). Our metabolic profiling identifies several alterations in lipid metabolism caused by either TDP^WT^ or disease associated TDP^G298S^; the accumulation of long-chain fatty acid carnitine conjugates and the decrease in carnitine point to defects in the carnitine shuttle. These defects may lead to detrimental effects on ATP production, since long-chain fatty acid import into the mitochondria is rate limiting for lipid beta oxidation. Consistent with this, beta-hydroxybutyrate (BHB), a ketone precursor resulting from lipid beta oxidation is significantly decreased in the context of both TDP^WT^ and TDP^G298S^ overexpression in motor neurons. These metabolite alterations provide a plausible explanation for the deficit in ATP production reported in ALS patients (Wiedemann et al., 2002). To further probe the involvement of the carnitine shuttle in motor neuron dysfunction we took a combined molecular, genetic and dietary approach. Transcriptional profiling experiments show that core components of the carnitine shuttle, CPT1, CPT2 and CACT are altered when TDP-43 proteinopathy is induced in motor neurons (see Figure 5 for model). The power of the *Drosophila* model is based in part on the ability to perform genetic interactions to probe the functional significance of transcriptional alterations in the carnitine shuttle. Indeed, reducing CPT1 levels in the context of TDP^WT^ overexpression in motor neurons by RNAi knock-down mitigates locomotor defects. Similarly, reducing CPT2 dosage in the context of TDP^G298S^ overexpression in motor neurons provides a similarly protective effect on locomotor activity. We note that since the qPCR experiments were conducted using whole VNCs, which comprise multiple cell types, our transcriptional profiling data is likely an underestimation of the actual molecular changes due to TDP-43 overexpression in motor neurons or glia specifically. Additionally, these results suggest that although TDP^WT^ and TDP^G298S^ cause overall similar ALS –like phenotypes, the underlying molecular mechanisms are variant specific and highlight the importance of understanding distinct types of ALS when designing therapeutic approaches.

Even though the mechanism underlying expression alterations of the carnitine shuttle remains to be determined, our findings strongly suggest complex interactions between TDP-43 and the carnitine shuttle major components. Our data support the hypothesis that carnitine conjugation and/or shuttling into the mitochondria is impaired in a TDP-43 dependent manner. Furthermore, dietary intervention with medium-chain fatty acids or BHB supports the idea that bypassing the carnitine shuttle provides alternative fueling sources for mitochondria in disease. In addition to the possibility of being direct targets of TDP-43, it is reasonable to suspect that abnormalities in mitochondrial morphology may contribute to carnitine shuttle deficits. Indeed, mitochondrial morphology has shown to be altered in a TDP-43 knock-in mouse (Stribl et al., 2014) and spinal cords from sporadic ALS patients (Sasaki and Iwata, 2007; Khalil et al., 2017). Furthermore, mitochondrial aggregates found in TDP mutant (TDP^M337V^) mice (Xu et al., 2011) may alter the expression and localization of carnitine shuttle components. Recently, TDP-43 was shown to localize to mitochondria and disease associated mutations enhance this association in primary motor neurons (Wang et al., 2013). Although these observations have not been confirmed in follow up studies in transgenic mice and patient fibroblasts (Onesto et al., 2016; Kawamata et al., 2017), a potential direct role for TDP-43 in mitochondrial homeostasis is an attractive scenario that requires further investigation. Recent reports showing that no bioenergetic defects are detectable in transgenic mice (TDP^A315T^) or patient fibroblasts (Kawamata et al., 2017) are seemingly discrepant from our findings, and remain to be resolved in future studies. Although it is possible that differences in models and approaches could explain this apparent discrepancy, the evidence of cellular energetics deficits in ALS is compelling (Dupuis et al., 2011; Schmitt et al., 2014).

Our findings that dietary intervention mitigates locomotor dysfunction in *Drosophila* is consistent with previous reports that heptanoate can improve the TCA cycle, albeit in a mouse model of epilepsy (Hadera et al., 2014). Heptanoate acts as an alternative neural fuel (Marin-Valencia et al., 2013), however, the fact that the liver can also metabolize it to ketones can confound its neuronal specific effects (Borges and Sonnewald, 2012). Our coconut oil experiments are also consistent with findings that high caloric diet, specifically, high fat had a positive effect on weight maintenance in ALS patients (Dorst et al., 2013). This is in contrast to a previous study showing that a high carbohydrate diet is beneficial and leads to less adverse effects than a high fat diet in patients (Wills et al., 2014). These contrasting findings highlight the challenge of devising a precise formula, comprising the right proportion of lipid types (e.g., medium and short-chain fatty acids), for dietary intervention in patients.

Finally, the positive effects of BHB feeding are consistent with a previous report that a ketogenic diet protects against motor neuron death in SOD1 mice (Zhao et al., 2006). The presence of high ketone levels in CSF or serum from ALS patients (Blasco et al., 2010; Kumar et al., 2010) suggests that in disease, metabolites may leak from the CNS. Taken together, our findings and published reports of metabolic dysregulation highlight the importance of reconciling cellular level with whole organism, system level metabolic studies, and the importance of model organism studies to increasing our understanding of cell type specific (e.g., motor neurons and glia), *in vivo* metabolic dysregulation associated with TDP-43 proteinopathy.

## Author contributions

EM, JB, and DZ designed the experiments. EM, AO, JB, DS, and GB performed experiments. EM, AO, JB, and DZ analyzed the data. EM and DZ wrote the manuscript.

### Conflict of interest statement

The authors declare that the research was conducted in the absence of any commercial or financial relationships that could be construed as a potential conflict of interest.
